# Body Contouring as Gender-Affirming Surgery in Transgender Patients: A Systematic Review of the Current Literature

**DOI:** 10.3390/jcm13123523

**Published:** 2024-06-16

**Authors:** Alejandra Aristizábal, María Ríos-Sánchez, Joseph M. Escandón, Dean DeRoberts, Enrique Armenta, Gabriel Del Corral, Andrés Mascaro, Oscar J. Manrique

**Affiliations:** 1 Division of Plastic Surgery, Department of Surgery, Mayo Clinic, Rochester, MN 55905, USA; 2 Department of Surgery, Mayo Clinic, Rochester, MN 55905, USA; 3 Syracuse Plastic Surgery, Syracuse, NY 13224, USA; 4 Department of Plastic Surgery, MedStar Georgetown University Medical Center, Washington, DC 20007, USA; 5 Department of Plastic and Reconstructive Surgery, Cleveland Clinic, Weston, FL 44195, USA

**Keywords:** gender-affirming body contouring, gender-affirming chest wall contouring, upper and lower body contouring, trans male body contouring, trans female body contouring

## Abstract

**Background:** There is an increasing demand for body contouring and gender-affirming surgeries, and so is the need to compare outcomes between techniques. Gender dysphoria is a discrepancy between gender identity and the sex assigned at birth. One way to address this is to perform procedures to enable patients to look according to their desired gender identity. Gaps in knowledge regarding the best approaches and which surgical techniques yield the most patient satisfaction remain. This article summarizes up-to-date studies, including upper and lower body contouring procedures. **Methods:** A systematic review was performed using terms related to body contouring in gender-affirming surgery for transgender patients. All articles included surgical and patient-reported outcomes following either chest or lower body contouring procedures. **Results**: 15 studies, including trans male chest wall contouring, trans female breast augmentation, and lower body contouring, with 1811 patients, fulfilled the inclusion criteria. The double incision (DI) techniques consistently resected more tissue and had better BODY Q scores than non-overweight patients. Bleeding was increased in periareolar, semicircular, and obese patients with DI techniques. Nipple depigmentation and sensation loss were more common with double-incision-free nipple graft techniques (DIFNG). Lower body contouring patients had average implant sizes bigger than 200 mL and reported 2 gluteal implant displacements, 1 exposure, and one rupture. Eight percent of patients who underwent large-volume fat grafting reported dissatisfaction due to fat reabsorption. Conclusions: The debate between the double incision and periareolar techniques continues. Variations of the DIFNG technique continue to be the most common approach; however, nipple depigmentation and loss of sensation are also more common with it. Regarding increased bleeding with periareolar techniques, there is still no evidence that hormonal therapy may be playing a role in it. For lower-body trans female contouring, implants could help with the longevity of contouring results in patients needing large-volume fat grafting. There is an increasing evaluation of gender-affirming body contouring patient-reported outcomes; however, there is still a need for a validated way to report satisfaction scores in lower body contouring. Validated surveys could help identify surgical candidates based on satisfaction patterns, specifically for transgender and non-binary patients.

## 1. Introduction

Gender-affirming procedures are becoming more common in the US due to increased awareness of gender dysphoria and expanding insurance coverage [1]. Efforts such as the “Affordable Care Act” have decreased barriers for the Lesbian, Gay, Transgender, Bisexual, Queer/Questioning, Intersexual, Asexual, and more (LGTBQIA+) community to access gender affirming surgeries (GAS) [2]. Gender dysphoria is a discrepancy between gender identity and the sex assigned at birth. One way to address this is to perform procedures to enable patients to look according to their desired gender identity [3,4]. Without mitigating factors such as a good support system, mental health assistance, hormone therapy, and surgery when desired, gender dysphoria can lead to drug abuse, depression, and other mental health issues that can ultimately end in suicide [2,5]. This is the rationale behind why gender-affirming care and gender-affirming surgeries should be considered a medical necessity for patients who fulfill the World Professional Association for Transgender Health (WPATH) standards [5,6]. 

Hormone therapy helps trans patients achieve soft tissue characteristics of the gender they identify with; however, if started after puberty, the change might not be to the extent that the patient desires [7,8]. This is why body contouring is an important aspect of gender dysphoria mitigation, allowing trans patients to overcome the incongruity between how they look and their gender identity [8]. Upper body contouring includes mastectomy for patients with developed breast tissue; its goal is to remove it, allowing trans males to stop binding their chests [9]. It also includes breast augmentation for patients who want a bigger chest profile than the one achieved with hormone therapy.

Lower body contouring is more common in trans female patients and consists of redistributing fat, putting implants, or both in the hip and buttock areas. Since hormone therapy does not change the bone structure of the pelvis, surgery is always needed to change these areas’ shapes [4,8,10].

Due to this increasing demand for body contouring gender-affirming surgeries and the fact that, when indicated, it is a treatment for gender dysphoria, the need to compare surgical outcomes between different techniques is exponentially rising to offer the best results [1,6]. There have been previous reviews about this topic, especially for chest wall contouring procedures in trans male patients [11], and since then, there has been an increase in the number of patient-reported outcomes (PROs) with validated surveys such as the BODY Q and others [12]. However, there are still some gaps in knowledge as to which are the best approaches and which types of surgical techniques yield the most patient satisfaction [3,6,7,8]. A systematic review was performed to evaluate the up-to-date studies, including upper and lower body procedures, that evaluate surgical outcomes, aesthetic results, and patient-reported outcomes specifically in transgender and non-binary patients [7].

## 2. Materials and Methods

This review followed the criteria of the Preferred Reporting Items of Systematic Review and Meta-Analysis (PRISMA) [13]. A systematic electronic search was performed from 3 April through 5 April 2024, in PubMed, Medline-Ovid, Embase, and Scopus. Terms related to body contouring in gender-affirming surgery and body contouring procedures in transgender patients were used (Supplementary File S1). There were no restrictions regarding date or language.

Two authors independently screened the title and abstract to determine the papers’ relevance for the full-text review phase. The author’s discussion resolved any differences regarding the inclusion of the articles. 

**Figure 1:**
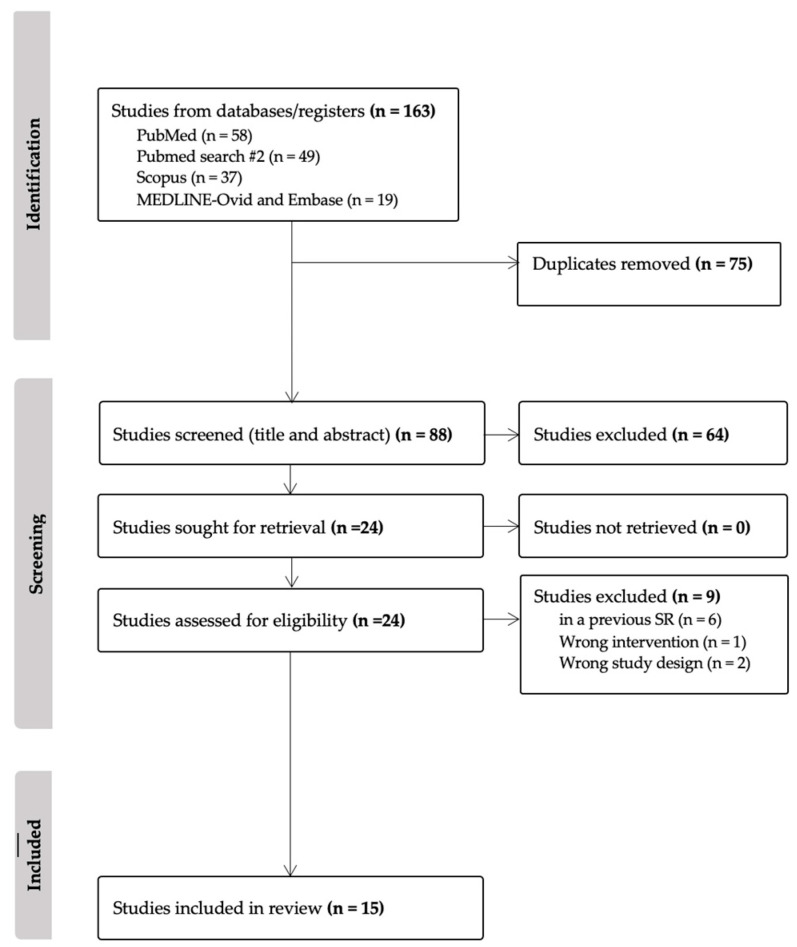
PRISMA diagram.

All articles included evaluated surgical and patient-reported outcomes following either chest or lower body contouring procedures in transgender patients. Articles with no transgender patients, abstracts, textbook chapters, case reports, review articles, and commentaries about previously published articles were excluded. The articles previously included in a 2018 systematic review about chest wall contouring surgery in trans male patients were also excluded from this review since the goal was to assess the current literature and not perform a quantifiable result, such as a meta-analysis with all the existing literature [12]. The cohort studies included were assessed for risk of bias with the New Castle Ottawa scale (NOS) by two independent authors (A.A., M.R.) Table 1, Figure 1 (Supplementary File S1). This systematic review was not registered as a protocol on PROSPERO. 

### Data Collection and Data Analysis

Two authors made an independent data extraction into an Excel spreadsheet with the variables of interest; these were presented according to the study. Continuous variables are presented as a mean with standard deviation, a median with range, or percentages (Table 2, Table 3, Table 4, Table 5 and Table 6). Any discrepancies were solved by the authors’ consensus. 

Variables such as surgical outcomes, complications, aesthetic outcomes, and patient-reported outcomes were collected and analyzed. 

## 3. Results

The literature search yielded a total of 163 articles, of which 75 were identified as duplicates. 88 titles and abstracts were screened; of these, 64 were considered irrelevant, and 24 full-text articles were screened. 15 studies with 1811 patients were considered to fulfill the inclusion criteria; 11 included 1486 trans male patients who underwent chest masculinization surgery, 1 included 109 trans female breast augmentation patients, and 3 included 216 trans female patients who got hip and buttock gender-affirming contouring. The studies included in the analysis were cohort studies and case series; no randomized controlled trials were included. The bias assessment using the NOS criteria categorized 7 studies as fair, with a score of 6 points, and 6 studies as good, with a score between 7 and 8 points. Due to the heterogeneity of outcomes reported by the studies, no statistical synthesis or meta-analysis was conducted to present effect measures of the outcomes.

Most chest-wall contouring studies were case series ranging from 11 to 464 patients. Nine articles compared outcomes from different mastectomy incisions in trans male patients, as well as aesthetic and patient-reported outcomes (PROs) [14,15,16,17,18,19,20,21,22], while two focused only on the latter [12,23].

The most reported incision was the conventional double incision mastectomy with free nipple graft (DIFNG) in 384 patients; in second place was the periareolar (P) incision in 251 patients; and the semi-circular incision (SC) in 73. The other double-incision mastectomy variations are listed in Table 2.

Four articles compared the conventional DIFNG versus a modification of this technique, and the other five compared the DIFNG to less invasive periareolar incisions and their modifications as well.

Only one article regarding breast augmentation in trans female patients was found, using submuscular round silicon prostheses [24] (Table 3).

As for the gluteal-hip contouring articles, they ranged from 11 to 172 patients, and all of them compared the use of liposuction, fat grafting, and either gluteal or hip silicone implants [10,25,26] (Table 3).

**Table 1:** Studies included.

Author	Study
Whitehead 2018 [19]	A Single Surgeon’s Experience with Transgender Female-to-Male Chest Surgery
Junn 2021 [14]	Hockey stick incision: a modified technique for chest wall masculinization
Decuypere 2020 [24]	Male-to-female gender affirmation surgery: breast reconstruction with Ergonomix round prostheses
Bustos 2020 [15]	The Nipple Split Sharing vs. Conventional Nipple Graft Technique in Chest Wall Masculinization Surgery: Can We Improve Patient Satisfaction and Aesthetic Outcomes?
Stein 2021 [17]	Surgical and patient-reported outcomes following double incision and free nipple grafting for female to male gender affirmation: does obesity make a difference?
Tamulevicius 2024 [16]	Subcutaneous mastectomy in female-to-male transsexuals is associated with higher risk of postoperative bleeding complications.
Sundhagen 2023 [18]	Chest Wall Contouring in Transgender Men: A 20-Year Experience from a National Center.
Elias 2022 [20]	Breaking the Binary: The Approach to Chest Masculinizing Gender-Affirming Surgery in Transgender Men
Makkonen 2024 [22]	Masculinizing chest-wall surgeries in transgender patients, a retrospective single-center study
Klassen 2021 [12]	The BODY-Q Chest Module: Further Validation in a Canadian Chest Masculinization Surgery Sample
Saarinen 2023 [23]	Quality of Chest Masculinization in Trans Men: A Retrospective Study Evaluating Surgical Technique, Complications, Secondary Corrections, and Trends
Kamali 2021 [21]	Improved Surgical Outcome with Double Incision and Free Nipple Graft in Gender Confirmation Mastectomy
Del Vecchio 2021 [10]	Body Feminization Combining Large-Volume Fat Grafting and Gluteal Implants
Cardenas-Camarena 2023 [26]	Aesthetic Augmentation of the Trochanteric Gluteal Region in Patients with Gender Dysphoria: Lipoinjection, Gluteal Implants, and Hip Implants
Cárdenas-Camarena 2020 [25]	Tridimensional Combined Gluteoplasty: Liposuction, Buttock Implants, and Fat Transfer

**Table 2:** Trans Male Contouring Studies.

Author	Intervention	Incision Type	Patients	Mean Age	BMI	Follow Up (Months)
Whitehead 2018 [19]	DIFNG vs. DINTP	DIFNG N = 20 DINTP N = 79	N = 99	DIFNG: 33.8 ** 22.1–63.5 DINTP: 33.0 17.3–60.6	DIFNG: 31.1 22.7–48.5 DINTP: 26.2 18.1–48.5	DIFNG: 3.7 1.0–29.0 DINTP: 7.3 1.0–114.4
Junn 2021 [14]	Compares HS, conventional DIFNG, periareolar	HS cohort 1 + 2 N = 14 DMFNG N = 6 Periareolar N = 6	N = 73 39 (53.4%) Answered survey 27 (37%) Provided complete data.	P: 18.8 ± 1.7 I: 25.8 ± 9.7 HS1: 30.1 ± 5.9 HS2 36.4 ± 5.5	Normal weight P: 6 (100%), I: 1 (16.7%) HS1: 3 (27.3%), HS2: 0 (0.0%) Overweight P 0 (0.0%), I: 1 (16.7%) HS1: 2 (18.2%), HS2: 0 Obese P 0 (0.0%), I: 4 (66.7%) HS1: 6 (54.5%), HS2: 4 (100%)	P: 166.2 ± 203.52 I: 73.2 ± 114.6 HS1: 205.5 ± 280.2 HS2: 53.0 ± 53.6
Bustos 2020 [15]	Conventional DIFNG vs. nipple split sharing	DIFNG N = 18, 36 Bs DINS N = 16, 32 Bs	N = 34	DINS: 27 (22–30) ** DMFNG: 24 (18–48)	DINS: 35.4 (22–38.2) DIFNG: 32.2 (23.1–45.3)	DINS: 13 (12–23) DIFNG: 11.5 (9–15)
Stein 2021 [17]	DIFNG obese vs. non-obese patients	DIFNG NO N = 54 DIFNG OB N = 43	N = 97	NO: 24 (6.1) OB: 29 (8.6)	NO: 25 (3.1) OB: 35 (5.9)	NO: 62 (12–112) OB: 61 (10–127)
Tamulevicius 2024 [16]	Compares incisions: CC, SC, IPMR, IMFNG	CC N = 10 SC N = 6 IPMR: N = 4 IMFNG: N = 24	N = 22	CC: 27.2 ± 7.8 IPMR 17.5 ± 2.1 IMFNG 22.4 ± 7.5 SC 63 ± 4.0	CC: 23.7 ± 1.7 IPMR 22.6 ± 0.8 IMFNG 27.8 ± 6.3 SC 24.1 ± 2.	NA
Sundhagen 2023 [18]	IFNG vs. periareolar	IFNG: N = 209 P: N = 124	N = 333	P: 20.2 (5.9) IFNG: 21.7 (8.5)	P: 22.4 (0.4) IFNG: 26.2 (0.3)	NA
Elias 2022 [20]	Compares: PNAC vs. OSR vs. SSSM vs. CMFNG	PNAC: N = 14 OSR: N = 2 SSSM: N = 38 CMFNG: N = 56	N = 110	PNAC: 24.6 ± 6.8 OSR: 26.5 ± 6.3 SSSM: 22.3 ± 6.3 CMFNG: 22.0 ± 5.9		NA
Makkonen 2024 [22]	Periareolar vs. double incision	P N = 8 DI N = 8	N = 16	P: 22.8 ± 3.10 DI: 27.5 ± 9.15	P: 21.7 ± 2.52 DI: 27.8 2.92	P: 90.3 46.1 DI: 69.1 31.9
Klassen 2021 [12]	BODY Q	no technique comparison	N = 115	mean range 26 (16–61)	27 (117.5–46.8)	Survey at 6 weeks and 6 months postop.
Saarinen 2023 [23]	BODY Q	no technique comparison	N = 123 respondents	median IQR 23 (7)	23.5 (6.2)	15 (19)
Kamali 2021 [21]	DIFNG vs. periareolar vs. semicircular	DIFNG N = 243, 52.4% P: N = 113, 24.4% SC: N = 67, 14.4%	N = 464	mean range 24.1 (14–64)	BMI < 18.5: 8 (1.7%) 18.5–24.9: 216 (46.6%) 25.0–29.9: 102 (22.0%) >30.0: 36 (7.8%) Missing: 102 (22.0%)	NA

Values given as mean/SD, If not otherwise stated, ** Median/range, Bs: Breasts, N: Normal weight, NO: non-obese, OB: obese, NA: no data available, HS: Hockey stick incision, NAC: Nipple areola complex, vs.: versus, DINTP: double incision with nipple transposition in a pedicle IFNG: inframammary incision with free nipple graft, DI: double incision, DIFNG: double incision with free nipple graft, DINS: double incision nipple split technique, P: periareolar, SC: semi-circular, I: inframammary HS: Hockey stick, CC: concentric circumareolar, IPMSR: inferior pedicle mammaplasty with skin resection, IMSRFNG: inframammary skin resection with full thickness free nipple graft, PNAC: Periareolar with superiorly based NAC flap, OSR: Omega-shaped resection, SSSM: Spindle-shaped simple mastectomy with NAC inferiorly based flap, CMFNG: Complete mastectomy with free NAC graft, IQR: interquartile range.

**Table 3:** Trans Female Contouring Studies.

Author	Intervention	Patients	Mean Age	BMI	Follow-Up (Months)
Cardenas Camarena 2020 [25]	Buttock implant placement, frame liposuction, lipoinjection in the lateral third of the buttock	N = 53 11 Trans female 41 cis female	33.3 23–49	NA	NA
Cardenas Camarena 2023 [26]	Trochanteric gluteal region liposuction + lipoinjection + gluteal + hip implant placement.	N = 172 1: N = 132 (76.7%) 2: N = 22, 12.7% 3. N =10 (10.4%) 4. N = 8	36.4 (23–56)	24.3 (21.2–27.1)	26 (3–45)
Del Vecchio 2021 [10]	Large-volume fat transplantation with or without gluteal implants.	36 Trans female F: N = 24 F + I: N = 12	F: 29.8 ± 6.0 F + I: 30.3 ± 7.0	F: 24.8 ± 1.8 F + I: 25.0 ± 2.0	Range (8–24)
Decuypere 2020 [24]	Breast augmentation: submuscular plane + inframammary incision	N = 109 Trans female	33.2 ± 14.6	23.6 ± 4.3	19.5 ± 7.9

Group 1: Liposuction + Lipoinjection Buttock + Lipoinjection Hips, Group 2: Liposuction + Gluteal Implants + Hip Lipoinjection, Group 3: GI + HI, Group 4: Liposuction + Gluteal Implants + Hip Implants + Lipoinjection. LI: lipo injection, LS: liposuction, GI: gluteal implant, HI: hip implant: H: hip, Buttock: B, sss: soft solid silicon, F: fat only, F + I: fat + implant, TA: transition area, OI: over implant, NA: no data available.

### 3.1. Surgical Outcomes 

For patients needing more than 1 kg to be resected, the inframammary resection, the Hockey Stick (HS) [14], the inframammary skin resection with full-thickness free nipple graft (ISFNG) [16], and the complete mastectomy with free NAC graft (CMFNG) [20] were suitable. The periareolar and semicircular techniques reported more bleeding, as did obese patients who underwent a DIFNG [16,17,18]. Nipple necrosis was reported as 0 in most studies; however, there were 7 cases in double incision patients, of which 4 were partial [15,19], 7 cases in periareolar, and 7 in inframammary incisions [18]. Dehiscence was more common in Class II (BMI 35–39.9) obese patients who had a double-incision mastectomy [17] and the first cohort of HS incisions [14]. As for the return to the operating room, the DINTP had the most patients, 31 (39.2%) [19], followed by periareolar incisions with a superiorly based NAC flap (PNAC), spindle-shaped simple mastectomy with an inferiorly based NAC flap [20] and periareolar techniques in Makkonen and Kamali’s study [21,22] (Table 4). 

For the trans female breast augmentation patients, the only complication reported was hematoma (0.46%) needing surgical evacuation in one patient [23]. Those who received gluteal implants had consistently larger implant sizes (>200 mL) and less fat transfer compared to those who did not receive implants. There was only one case of implant exposure [25]. There were two hip implant displacements and one implant rupture; this study was the only one to report fat reabsorption and seroma rate [26] (Table 5). 

**Table 4:** Trans male chest contouring surgical outcomes.

Author	Specimen Weight	Bleeding	Seroma	Hematoma	Infection	NAC Necrosis	Fat Necrosis	Dehiscence	RTOR
Whitehead 2018 [19]	DIFNG: 872.0 198.0–1908 DINTP: 398.0 116.0–1481	NA	DIFNG: 1 (5.0) DINTP: 2 (2.5)	DIFNG: 1 (5.0) DINTP: 2 (2.5)	NA	DIFNG 0 DINTP: 1 (1.0)	NA	NA	DIFNG: 6 (30.0) DINTP: 31 (39.2)
Junn 2021 [14]	P: 320.8 ± 191.5 I: 1769.5 ± 946.8 HS1: 2030.7 ± 1036.9 HS2: 3623.7 ± 1312.9	NA	P: 1 = 16.7% I: 1 = 16.7% HS1: 1 HS2: 0	P: 2 (33.3%) I: 0% H: 0 HS2: 0	P: 0 I: 1 (16.7%) H: 2 (18.2% ) HS2: 0	P: 0% I: 0 H: 0 HS2: 0	P: 0% I: 0 H: 0 HS2: 0	P: 0% I: 1 (16.4%) HS1: 4 (36.4%) HS2: 2 (50%)	P: 0% I: 0 H: 0
Bustos 2020 [15]	DINS: 750 g (85–1.000) DIFNG: 820 g (100–1.515)	NA	DIFNG: 0 DINS: 0	DIFNG: 0 DINS: 0	DIFNG: 0 DINS: 0	DIFNG: 0 DINS: 0 Partial DIFNG: 4 DINS: 0	NA	DIFNG: 1(2.8%) DINS: 0	NA
Stein 2021 [17]	NO: 464 (270), R: 445 (255) OB: L: 961 (553), R 919 (522)	NO: 62.0 (84.2) OB: 113 (100)	NO: 0 OB:2 (2.6)	NO: 0 OB:1 (2.3)	NO:3 (5.6) OB:1 (2.3)	NO: 0 OB: graft loss = 1 2.3%	NA	NO: 0 OB: 3 (7.0)	NA
Tamulevicus 2024 [16]	CC: 201.2 ± 191.6 IPM 416.5 ± 84.2 ISFNG 1167.9 ± 662.0 SC 200.7 ± 135.5	CC: 1 (10.0%) IPM: 1 (25%) ISFNG 5 (20.8%) SC 2 (33.3%)	N = 1, 2.3%	NA	NA	0 reported	NA	grouped with seroma	NA
Sundhagen 2023 [18]	NA	P: 17 (13.7) I: 15 (7.2)	P: 4 (3.2) I: 3 (1.4)	P: NA I: NA	P: 6 (4.8) I: 7 (3.3)	P: 7 (5.6) I: 7 (3.3)	P: NA I: NA	P: 1 (0.8) I: 2(1.0)	
Elias 2022 [20]	PNAC: 485 ± 362 OSR: 310 ± 56 SSM:681 ± 394 CMFNG: 1157 ± 853	NA	NA	PNAC: 0 OSMR: 0 SSSM: 3, 7.9% CMFNG: 3, 5.3%					PNAC: 7 (50%) OSR: 1 (50%) SSSM: 7 (50%) CMFNG: 3 (5.3%)
Makkonen 2024 [22]	NA	P: NA D: NA	P: 0 D:1 (1.11)	P: 1 (1.11) D:1 (1.11)	P: 1 (1.11) D:2 (2.22)	P: 0 D:1 (1.11)	P: NA D: NA	P: NA D: NA	P: 6 (85.7) D:3 (33.3)
Klassen 2021 [12]	NA		N = 7	N = 8		N = 5			
Saarinen 2023 [23]	NA	NA	NA	NA	NA	NA	NA	NA	NA
Kamali 2021 [21]	NA	NA	NA	NA	NA	NA	NA	NA	DIFNG: 8 (3.3%) P: 14 (12.4%) SC: 8 (11.9%) Other techniques 33 (7.3%) Total N = 33 (7.1%)

RTOR: return to the Operating Room, NA: no data available.

**Table 5:** Male to Female Contour surgical outcomes.

Author	Implant Size mL	Implant Issues	Fat Transfer Volume	Fat Reabsorption	Seroma	Dehiscence	RTOR	Other
Cardenas Camarena 2020 [25]	258.1 (100–400)	Implant exposure: 1 (1.9)	Mean range Hip: 258.1 100–400 TA: 141.6 50–200 OI: 137.5 100–175	NA	NA	Partial: 6 (11.5)	For revision: 1 (1.9)	NA
Cardenas Camarena 2023 [26]	1. No implant 2. 210–450 (310) 3. GI: 180–400 (290) HI: 180–250 (220) 4. GI: 200–420 (280) HI: 180–230 (200)	1. No implant 2. 1 asymmetry 3. 2 HI displacements, 1 implant removal, 1 implant rupture 4. None	Volume per side 1. 220–630 (423) 2. 220–390 (sss318) 3. Not applicable 4. 80–120 (105)	1.10 (7.5) 2. 3 (13.6) 3. not applicable 4. 0 (0)	1. 0 2. 0 3. 0 4. 1 (12.5) sss	Gluteal dehiscence 1. not applicable 2. 3 (13.6) 3. 1 (10) 4. 0	NA	Satisfaction 1. 122 (92.4) 2. 18 (81.8) 3. 6 (60) 4. 8 (100) Dissatisfaction: 4 of 10 subfascial plane implant
Del Vecchio 2021 [10]	N = 12 intramuscular implants Due to not enough fat available. round cohesive gel implants, 270 (235–335)	NA	Mean transplanted per buttock mL:1100 Average harvested 2.700 mL	NA	NA	NA	Second round fat grafting N = 4 no group specified	Waist-hip ratio Preop F: 1.1 ± 0.1 F + I: 1.1 ± 0.1 Post op F: 0.88 ± 0.06 F + I: 0.75 ± 0.06
Decuypere 2020 [24]	402 ± 70 cc	0	No fat grafting	No fat grafting	0	0	Hematoma N = 1 Surgical drainage	Satisfaction: with breasts N = 51 76.8 ± 18.9 with outcome 74.8 ± 21.8 with implants 7.0 ± 1.5 Physical well-being: chest 85.9 ± 17.4

Bleeding, hematoma, infection, fat necrosis, and scar issues were not mentioned in the articles. Group 1: Liposuction + Lipoinjection Buttock + Lipoinjection Hips, Group 2. Liposuction + Gluteal Implants + Hip Lipoinjection, Group 3. GI + HI, Group 4: Liposuction + Gluteal Implants + Hip Implants + Lipoinjection. LI: lipo injection, LS: liposuction, GI: gluteal implant, HI: hip implant: H: hip, Buttock: B, sss: soft solid silicon, F: fat only, F + I: fat + implant TA: transition area, OI: over the implant, NA: no data available.

### 3.2. Aesthetic and Patient-Reported Outcomes

Excess skin resection was the most common revision procedure, performed most frequently in patients with the DINTP incision [19], followed by the inframammary and periareolar techniques in the Sundhagen et al. study [18] which reported the highest rate of revisions in general; Klassen’s study did not report complications stratified by incision type [12]. Nipple depigmentation was more common in a double-incision mastectomy with free nipple grafts [15,19]. Only one study evaluated the sensitivity return in patients who underwent DINTP [19]. Two studies reported aesthetic scores [15,20], and six studies reported PROs using the BODY Q scale [12,14,15,17,22,23] (Table 6).

For breast augmentation, PROs were reported using the BREAST Q survey (Table 5). 

**Table 6:** Aesthetic and Satisfaction Outcomes Trans male chest wall contouring.

Author	Excess Skin Revision	Healing/Scar Issues	Other	Aesthetic Scores	Body Q Chest	Body Q Nipple	Overall Score
Whitehead 2018 [19]	Dog ear excision: DIFNG: 2 (10.0) DINTP: 23 (29.1)	Problematic scarring: DIFNG: 4 (20) DINTP: 13 (16.5) Steroid injection: DMFNG: 3 (15) DINTP: 5 (6.3) Revision: DMFNG: 2 (2.0) DINTP: 0	NAC depigmentation DIFNG: 6 (30.0) DINTP: 6 (7.6) Nipple sensation DINTP: 94.1% at least some nipple sensation. 66.2% = full bilateral 20.6% = some bilateral 7.4% = some unilateral 5.9% = no sensation Not evaluated for DIFNG	NA	DIFNG: NA	DIFNG: NA	DIFNG: NA
Junn 2021 [14]	NA	Scar revision None in all groups	POP complications by BMI N: N = 10, C = 4 OV: N = 3, C: 1, OB: N = 14, C = 8	NA	P: 67.33 ± 27.35 I: 62.83 ± 34.45 HS1:89.6 ± 11.9, HS2: 72.0 ± 24.7 by BMI Chest: N: 79.70 ± 25.98 OV: 62.33 ± 54.37 OB: 76.50 ± 17.19	P: 63.00 ± 27.12 I: 68.33 ± 39.33 HS1:86.8 ± 15.9 HS2: 74.3 ± 25.3 by BMI Nipples: N: 74.60 ± 26.80 OV: 66.67 ± 57.74 OB: 78.14 ± 19.72	P: 645.17 ± 65 I: 657.67 ± 178.55 HS1: 722.7 ± 63.8 HS2: 669.3 ± 33.7
Bustos 2020 [15]	NA	DMFNG:1 DINS: 1 (3.1%)	Nipple depigmentation DIFNG: 2 (5.6%) DINS: 0	AIS scores DIFNG: 3.4 (1–5) DINS: 3.9 (1–5)	DMFNG: 75.8 (40–100) DINS: 84.3 (83–87)	Nipple → *p* < 0.0001 DMFNG: 58.1 (0–90) DINS: 90.7 (82–100	NA
Stein 2021 [17]	NA	NA	NA	NA	Mean/SD N: 89 (11) O: 84 (13)	N: 74 (19) O: 74 (18) non-*p* < 0.001	N:63 (22) O: 65 (18)
Tamulevicius 2024 [16]	NA	NA	NA	NA	NA	NA	NA
Sundhagen 2023 [18]	P: 7 (5.6) I: 14 (6.7)	P: 24 (19.4) I: 3 (14.4) Revisions P: 39 (31.5) I: 44 (21.1)	Lipo revision P: 13 (10.5) I: 8 (3.8)	NA	NA	NA	NA
Elias 2022 [20]	NA	NA	Revisions: PNAC: 7(50%) OSR: 1(50%) SSSM: 4(10.5%) CMFNG: 3 (5.3%)	PNAC 2.5 ± 1.55 OSR: 1.4 ± 0.71 SSSM: 4.19 ± 0.75 CMFNG:4.07 ± 1.00	NA	NA	NA
Makkonen 2024 [22]	NA	Secondary aesthetic corrections P: 5, 71.4 DI: 3 (33.33)	NA	NA	N = 2 DI and N = 4 P P: 47 D: 85	P: 63 D: 67	NA
Klassen 2021 [12]	Dog ear: N = 9	Delayed wound healing: N = 5	Complications not specified: 0: N = 87 (79.1%) 1: N = 17 (15.5%) >1: N = 6 (5.5)	NA	Scores presented in graphics	Scores presented in graphics	NA
Saarinen 2023 [23]	NA	NA	NA	NA	NA Scores displayed in graphics	NA	NA
Kamali 2021 [21]	NA	NA	Complications: not specified DI: 46 (18.9%) P: 32 (28.3%) SC: 13 (19.4%) other techniques: 8 (19.5%)	NA	NA	NA	NA

Scarring issues were consistently reported in Sundhagen´s and Whitehead’s studies. For the conventional DIFNG, there were fewer scar treatments. As for aesthetic scores, the spindle-shape simple mastectomy and the complete mastectomy with free NAC had the best scores compared to the conventional DINS and conventional DIFNG [15,20]. In general, patients with double incision techniques reported better BODY Q scores than normal-weight patients [14,15,22].

## 4. Discussion 

This updated systematic review gathered gender-affirming body contouring studies assessing surgical, aesthetic, and patient-reported outcomes (PROs). Trans-male chest wall contouring studies from the last 7 years continue to compare the outcomes of double incision mastectomies with free nipple grafts (DIFNG) to their different variations or periareolar techniques. As for lower contouring, the debate persists between using implants, fat grafting, or both. There has been an increase in the report of aesthetic and patient-reported outcomes in body contouring-affirming surgery [12]. 

The BODY Q survey is a PRO instrument that measures health-related quality of life after body contouring or weight loss. The chest module of this survey was developed to assess the appearance of the chest and nipple [12]. The BODY Q chest module was reported in six trans-male studies. The BREAST Q, another instrument that assesses different patient-reported outcomes in breast surgery, was reported in a rrans female chest-wall study [24,27]. Two studies compared the BODY Q chest scores between double incision techniques and periareolar, with higher scores in the first technique [14,22]. There were no statistically significant differences between obese and non-obese patients’ scores [14,17]. This brings up a discussion about whether patients prefer the flatter chest that the double incision techniques allow by enabling more tissue and skin resection, despite the larger scars. It is important to discuss this with the patient, even when they appear to be candidates for one of the two approaches, as the general rule is that a double incision technique is needed for larger breast tissue, while smaller breasts can be addressed with periareolar techniques [11,18]. Since these surgeries aim to mitigate gender dysphoria, the need to assess patient satisfaction is very important [5,6,8].

Despite the increasing body contouring procedures, there is no validated scale to report PROs in gender-affirming lower body contouring; however, its outcomes have gained more importance and are being reported with non-validated surveys [26]. 

Variations of the double incision mastectomy with free nipple graft continue to be the most common approach and are better at addressing large-volume mastectomies, resulting in less revision liposuction and excess skin resection despite causing a noticeable scar [21]. The least invasive periareolar approaches report more bleeding [18]. These findings are consistent with a previous systematic review by Cohen et al. [11]. However, there is still no evidence that hormonal therapy may be playing a role in this, and it is still attributed to the smaller visual field available. However, the studies do not consistently report the use of hormonal therapy by their patients, making it difficult to assess how this could affect postoperative outcomes, especially regarding bleeding and thromboembolic events [16]. 

Nipple-areolar complex necrosis is similar among both approaches; although nipple depigmentation and loss of sensation are more common in double-incision mastectomies, this plays an important role in patient satisfaction, prompting a careful discussion about nipple sensation and appearance after DI mastectomies with the patient [28]. In contrast to previous literature, nipple sensation was assessed in patients with double incisions with nipple transposition on a pedicle, reporting a 94% return of at least some sensation; nonetheless, there is still more research to do about this outcome with this technique [19]. 

One of the difficulties encountered while analyzing the articles is that oftentimes complications and revision surgeries are not listed individually, or the articles do not specify the cause behind them by pooling together all complications and revision surgeries, making it difficult to evaluate which approach is better [21]. This could be due to their retrospective nature and the heterogeneity of possible revision causes [8,15,18]. 

As for the lower body contouring techniques, Camarena. et al. shed some light on the utility of using both implants and fat grafting [26]. Overall satisfaction was 89%, and in the only fat-grafting group, it was 92%. The remaining patients reported dissatisfaction due to fat reabsorption, in which case implants could help with the longevity of contouring results [26]. The rationale behind this could be due to the large volume of fat needed to mold the masculine osseous structure into a more feminine one, so this should be assessed accordingly with the patient´s body mass index and total body fat [10,25,26]. This highlights the importance of a validated survey to assess satisfaction in this patient population.

Due to the staggering increase in gender-affirming body contouring, it is important to report surgical, aesthetic, and patient satisfaction outcomes to further assess the current techniques and tailor them to patients’ needs. This study found the increasing evaluation of trans male and trans female body contouring techniques, patients’ satisfaction with instruments like the BODY Q survey, and the need for a validated way to report satisfaction scores in lower body contouring patients. The limitation of this study is that it is a systematic review of the literature, it was not registered in PROSPERO before its completion, and some articles could have been missed during the search, as well as the retrospective nature of the studies included. Most of the studies are small case series, and there is a low response rate in the studies that used surveys [14,22]. However, there is still a gap in the literature about complication rates, aesthetic results, and patient-reported outcomes in gender-affirming body contouring, and hopefully, this study sheds some light and inspires more research on the topic.

## 5. Conclusions

The reporting of surgical and patient outcomes in gender-affirming contouring surgeries is promising. This is needed to improve patient-centered care as well as patient satisfaction. Validated patient-reported outcomes surveys could help identify surgical candidates based on satisfaction patterns specifically for transgender and non-binary patients.

## Data Availability

Data is displayed in the article and the Supplementary Materials.

## Supplementary Materials

The following supporting information can be downloaded at: https://www.mdpi.com/article/10.3390/jcm13123523/s1.
