# Supplementary material for: Body Contouring as Gender-Affirming Surgery in Transgender Patients: A Systematic Review of the Current Literature
Source: J Clin Med. 2024 Jun 16;13(12):3523. doi: 10.3390/jcm13123523 (PMC11204619; doi:10.3390/jcm13123523)
Supplement: Supplementary file 1 [file jcm-13-03523-s001.zip › jcm-3015168-supplementary.pdf]

Systematic search:
Body contouring as gender-affirming surgery in transgender patients: A systematic review of the current literature**.**

1. **Pubmed:**
   Date of search: April 3rd /2024
   Database limit: no database limit has been applied.
   Search terms: non-controlled vocabulary and Boolean operators.

Pubmed:	Concept	Search string #	Search query	Results retrieved
pubmed	(body contour) AND (transgender)	#1	"human body"[MeSH Terms] OR ("human"[All Fields] AND "body"[All Fields]) OR "human body"[All Fields] OR "body"[All Fields]) AND ("contour"[All Fields] OR "contour s"[All Fields] OR "contoured"[All Fields] OR "contouring"[All Fields] OR "contours"[All Fields]) AND ("transgender persons"[MeSH Terms] OR ("transgender"[All Fields] AND "persons"[All Fields]) OR "transgender persons"[All Fields] OR "transgender"[All Fields] OR "transgendered"[All Fields] OR "transgenders"[All Fields])<br>**Translations**<br>**body:** "human body"[MeSH Terms] OR ("human"[All Fields] AND "body"[All Fields]) OR "human body"[All Fields] OR "body"[All Fields]<br>**contour:** "contour"[All Fields] OR "contour's"[All Fields] OR "contoured"[All Fields] OR "contouring"[All Fields] OR "contours"[All Fields]<br>**transgender:** "transgender persons"[MeSH Terms] OR ("transgender"[All Fields] AND "persons"[All Fields]) OR "transgender persons"[All Fields] OR "transgender"[All Fields] OR "transgendered"[All Fields] OR "transgenders"[All Fields]	36

Pubmed: All fields	Body contouring in gender affirming surgery		("body contouring"[MeSH Terms] OR ("body"[All Fields] AND "contouring"[All Fields]) OR "body contouring"[All Fields]) AND ("sex reassignment surgery"[MeSH Terms] OR ("sex"[All Fields] AND "reassignment"[All Fields] AND "surgery"[All Fields]) OR "sex reassignment surgery"[All Fields] OR ("gender"[All Fields] AND "affirming"[All Fields] AND "surgery"[All Fields]) OR "gender affirming surgery"[All Fields]) **Translations** **body contouring:** "body contouring"[MeSH Terms] OR ("body"[All Fields] AND "contouring"[All Fields]) OR "body contouring"[All Fields] **gender affirming surgery:** "sex reassignment surgery"[MeSH Terms] OR ("sex"[All Fields] AND "reassignment"[All Fields] AND "surgery"[All Fields]) OR "sex reassignment surgery"[All Fields] OR ("gender"[All Fields] AND "affirming"[All Fields] AND "surgery"[All Fields]) OR "gender affirming surgery"[All Fields]	22
	**(((((((transgender) OR (transfemale)) OR (transmale)) OR (binary)) OR (transwomen)) OR (transmale)) OR (transmen)) OR (transwoman)**	#3		117,123
	**(((((((gluteoplasty) OR (lower body contouring)) OR (hip augmentation)) OR (gluteal augmentation)) OR (chest wall contouring)) OR (upper body contouring)) OR**	#4		4.541

	**(body contouring)) OR (body contouring gender affirming surgery)**			
	**#3 AND #4**		"gluteoplasty"[All Fields] OR (("lower"[All Fields] OR "lowered"[All Fields] OR "lowering"[All Fields] OR "lowerings"[All Fields] OR "lowers"[All Fields]) AND ("body contouring"[MeSH Terms] OR ("body"[All Fields] AND "contouring"[All Fields]) OR "body contouring"[All Fields])) OR (("hip"[MeSH Terms] OR "hip"[All Fields]) AND ("augment"[All Fields] OR "augmentation"[All Fields] OR "augmentations"[All Fields] OR "augmented"[All Fields] OR "augmenting"[All Fields] OR "augments"[All Fields])) OR (("gluteal"[All Fields] OR "gluteals"[All Fields]) AND ("augment"[All Fields] OR "augmentation"[All Fields] OR "augmentations"[All Fields] OR "augmented"[All Fields] OR "augmenting"[All Fields] OR "augments"[All Fields])) OR (("thoracic wall"[MeSH Terms] OR ("thoracic"[All Fields] AND "wall"[All Fields]) OR "thoracic wall"[All Fields] OR ("chest"[All Fields] AND "wall"[All Fields]) OR "chest wall"[All Fields]) AND ("contour"[All Fields] OR "contour s"[All Fields] OR "contoured"[All Fields] OR "contouring"[All Fields] OR "contours"[All Fields])) OR (("upper"[All Fields] OR "uppers"[All Fields]) AND ("body contouring"[MeSH Terms] OR ("body"[All Fields] AND "contouring"[All Fields]) OR "body contouring"[All Fields])) OR ("body contouring"[MeSH Terms] OR ("body"[All Fields] AND "contouring"[All Fields])	49

			OR "body contouring"[All Fields]) OR (("body contouring"[MeSH Terms] OR ("body"[All Fields] AND "contouring"[All Fields]) OR "body contouring"[All Fields]) AND ("sex reassignment surgery"[MeSH Terms] OR ("sex"[All Fields] AND "reassignment"[All Fields] AND "surgery"[All Fields]) OR "sex reassignment surgery"[All Fields] OR ("gender"[All Fields] AND "affirming"[All Fields] AND "surgery"[All Fields]) OR "gender affirming surgery"[All Fields]))) AND ("transgender persons"[MeSH Terms] OR ("transgender"[All Fields] AND "persons"[All Fields]) OR "transgender persons"[All Fields] OR "transgender"[All Fields] OR "transgendered"[All Fields] OR "transgenders"[All Fields] OR "transfemale"[All Fields] OR "transmale"[All Fields] OR ("binaries"[All Fields] OR "binary"[All Fields]) OR "transwomen"[All Fields] OR "transmale"[All Fields] OR "transmen"[All Fields] OR "transwoman"[All Fields])	

Total first Pubmed search: 58
Second pubmed search: 49.

2. **Ovid Medline (R), Embase**
   Date of search: April 5th/2024
   Database limit: no database limit has been applied.
   Search terms: non-controlled vocabulary, and Boolean operators.

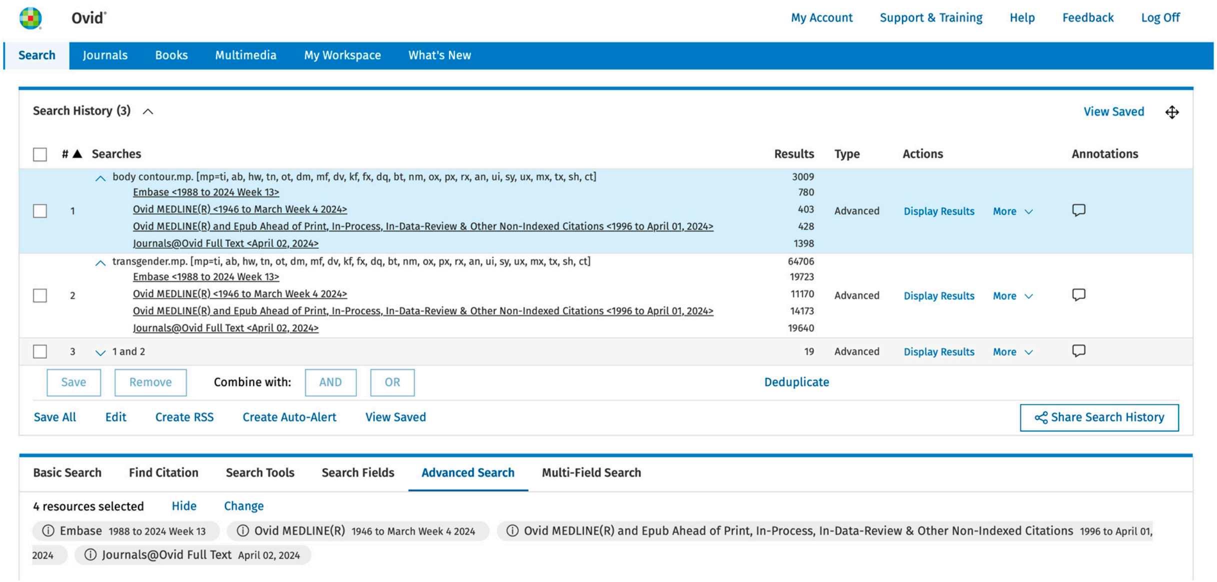

Total: 19 articles

3. **Scopus**
   Date of search: April 5th /2024
   Database limit: no database limit has been applied.
   Search terms: non-controlled vocabulary, and Boolean operators.

Scopus	Concept	Search string #	Search query	Results retrieved
	Body contouring and gender affirming surgery	#1	TITLE-ABS-KEY (body AND contouring) AND gender AND affirming AND surgery 37	37
